# Andrea Prudente case: a case study on social mobilisation, reproductive governance, and the politics of abortion reform in Malta

**DOI:** 10.1080/26410397.2026.2697378

**Published:** 2026-07-29

**Authors:** Andreana Dibben, Natalie Psaila Stabile, Isabel Stabile

**Affiliations:** a Senior Lecturer, Department of Social Policy and Social Work, University of Malta, Msida, Malta. *Correspondence:* andreana.dibben@um.edu.mt; b PhD Candidate, Department of Gender and Sexualities, University of Malta, Msida, Malta; c Professor, Department of Dental Surgery, University of Malta, Msida, Malta

**Keywords:** Malta, abortion, reproductive governance, legal reform, social mobilisation

## Abstract

In 2022, the case of Andrea Prudente, an American tourist denied an abortion for a non-viable pregnancy in Malta, catalysed unprecedented social mobilisation and compelled a legislative review of the country’s abortion ban. This paper traces the political and social dynamics that shaped this process, from the initial advocacy to the final, heavily contested legislative outcome. Drawing on Morgan and Roberts’ concept of reproductive governance, we examine media content and parliamentary discourses to trace how state actors mediated the conflict between competing moral and legal frameworks. Our analysis demonstrates that while persistent pressure by pro-choice advocates was instrumental in forcing a legislative review, the government’s initial proposed reforms were systematically contested and ultimately neutralised by a powerful, well-organised anti-choice countermovement. We argue that state actors ultimately capitulated to immense conservative pressures from influential religious and civil society actors. The resulting amendment, which permits termination only under exceptionally restrictive circumstances to save a pregnant person’s life, is a stark example of how reproductive governance can accommodate pressure for change while leaving the underlying system intact. This process highlights how legal reform can be critically decoupled from meaningful access, failing to secure genuine reproductive rights. Despite its profound limitations, the amendment constitutes the first-ever legal exception to Malta’s absolute ban, proving that such entrenched governance frameworks are not immutable. The Prudente case and the mobilisation it inspired have irrevocably fractured the previous politico-legal consensus, thereby creating a precedent for future legal challenges and continued advocacy for comprehensive reproductive justice in Malta.

## Plain language summary

In 2022, an American tourist in Malta named Andrea Prudente needed an abortion for a pregnancy that could not survive. Doctors refused because of the country's total ban. Her case sparked major protests and forced the Maltese government to review its abortion law. This paper studies how this process unfolded. We looked at newspapers and political speeches. We wanted to understand the arguments of those fighting for abortion rights and those against them. We found that those fighting for abortion pushed the government to act. But a powerful and well-organised anti-choice movement fought against any changes. The government gave in to pressure from influential conservative and religious groups. The result was a new law that is very restrictive. It only allows an abortion in rare cases to save the pregnant person's life. This change does not provide meaningful access to abortion care. Yet, this is the first time there has been a change to Malta's complete ban. This shows that the old rules can change. It creates an important starting point for future work to secure full reproductive rights in Malta.

## Résumé

En 2022, l’affaire Andrea Prudente, une touriste américaine qui s’est vu refuser un avortement à Malte en raison d’une grossesse non-viable, a catalysé une mobilisation sociale sans précédent et a contraint le pays à réviser la loi interdisant l’interruption de grossesse. Cet article retrace les dynamiques politiques et sociales qui ont façonné ce processus, depuis le plaidoyer initial jusqu’au résultat législatif final, fortement contesté. Nous fondant sur le concept de gouvernance reproductive de Morgan et Robert (2012), nous examinons les contenus médiatiques et les discours parlementaires afin de retracer comment les acteurs étatiques ont géré le conflit entre les cadres moraux et juridiques concurrents. Notre analyse démontre que si la pression persistante des défenseurs du droit à l’avortement a été déterminante pour imposer une révision législative, les réformes initialement proposées par le Gouvernement maltais ont été systématiquement contestées et finalement neutralisées par un puissant mouvement anti-avortement bien organisé. Nous affirmons que les acteurs étatiques ont finalement cédé face aux immenses pressions conservatrices exercées par des acteurs influents de la société civile et des milieux religieux. L’amendement en résultant, qui n’autorise l’interruption de grossesse que dans des circonstances exceptionnellement restrictives pour sauver la vie de la personne enceinte, illustre de manière frappante comment la gouvernance reproductive peut répondre aux pressions en faveur du changement sans modifier le système sous-jacent. Ce processus met en lumière le risque de dissociation critique entre réforme juridique et accès effectif, empêchant ainsi la garantie de véritables droits reproductifs. En dépit de ses profondes limitations, cet amendement constitue la toute première exception légale à l’interdiction absolue en vigueur à Malte, prouvant que de tels cadres de gouvernance profondément enracinés ne sont pas immuables. L’affaire Prudente et la mobilisation qu’elle a suscitée ont irrévocablement fracturé le consensus politico-juridique antérieur, créant ainsi un précédent pour de futurs recours juridiques et la poursuite du plaidoyer en faveur d’une justice reproductive complète à Malte.

## Introduction

In June 2022, the Family Planning Advisory Service (FPAS) helpline received a call from Jay Weeldryer, an American tourist. His partner, Andrea Prudente, had been hospitalised in Malta at 16 weeks of pregnancy. Prudente was experiencing a placental abruption and premature preterm rupture of membranes (pPROM). Since a fetal heartbeat was still detectable, the medical team at the hospital informed the couple that the standard protocol was to wait for fetal demise before any further action could be taken. The FPAS helpline referred the case to Doctors for Choice Malta (DFCM). Recognising the serious risk to Prudente's health, and that Malta’s absolute ban on abortion would prevent a necessary termination, the doctors at DFCM advised the couple to contact their travel insurance immediately to facilitate an emergency medical evacuation to another country.^1^ The couple described facing significant institutional resistance when attempting to obtain medical records for the insurance company. The hospital's medical team maintained that a termination was not clinically indicated and that the patient was receiving the best possible care.^2^ Faced with clinical and administrative barriers, the couple, assisted by pro-choice activists, brought their case to public attention. Ms Prudente was eventually flown by air ambulance to Spain, where she received the essential health care she needed.^3^

Landmark reproductive rights cases are recognised catalysts for legal reform, capable of reshaping abortion law by prompting judicial action, public debate, and legislative pressure. Studies across different countries (e.g. Ireland, Argentina, and Colombia) confirm that such cases can serve as turning points for policy change, precipitating direct legislative amendments or sustained discourse leading to subsequent reforms.^4–6^ The most direct parallel is the 2012 case of Savita Halappanavar in Ireland, who was similarly denied a termination during an inevitable miscarriage because a fetal heartbeat was detectable.^7^ Her subsequent death from septicaemia became the catalyst that re-ignited Ireland’s political debate.^7^ Both Andrea Prudente’s and Savita Halappanavar’s cases triggered a transition from unwritten medical protocols to exceptionally narrow legislation. In both contexts, the resulting laws were criticised for being so restrictive that they largely codified the existing chilling effect rather than resolving it.

Within this context, this paper analyses the sociopolitical dynamics and policy responses that emerged in Malta following the Andrea Prudente case, drawing on the theoretical framework of reproductive governance. As defined by Morgan and Roberts,^8^ reproductive governance refers to the methods through which various actors, including the state, religious institutions, financial bodies, non-governmental organisations, and social movements, use legislative, economic, moral, coercive and ethical mechanisms to shape population and reproductive policies.

### Maltese sociopolitical context

The island nation of Malta, the European Union’s smallest and southernmost member state, enforces some of the most restrictive abortion laws in Europe and worldwide. Up until 2023, the Maltese criminal code imposed a blanket ban on abortion, mandating a prison sentence of up to three years for a person who “induces a miscarriage” for “whatsoever reason”. For medical practitioners or other individuals who assist in the procedures, the penalties include up to four years’ imprisonment and the permanent loss of their professional license^9^ (ss. 241–243).

Malta gained independence from Britain in 1964 after 164 years of colonisation. The legal prohibition on abortion that originated in the British colonial-era criminal code persisted following independence. Malta’s nation-building project was deeply intertwined with the Catholic Church, which had, throughout centuries of foreign rule, positioned itself as a unifying force and the guardian of national morality and tradition.^10^ Its status was enshrined in the Constitution, which declares Roman Catholicism as the state religion and grants the Church the “duty and the right to teach which principles are right and which are wrong”.^11^ This constitutional position has historically allowed the Church to wield considerable influence over legislation and public life, ensuring that national laws align with its doctrines.^12^

This fusion of religious authority with post-independence identity is a recognised feature of postcolonial nation-building. As Fischer^13^ argued in the parallel case of Ireland, this process creates an affective politics of place, where the nation’s physical territory becomes symbolically conflated with a space of moral purity. In Malta, as in Ireland, a policy of “gendered displacement”^13^ has meant that women needing abortions were forced to travel abroad. On average, 42 Maltese residents accessed abortion services in England, Wales, and Spain, each year between 2016 and 2023.^14^ The phenomenon of compelled travel imposes significant burdens, including financial costs, time off from work, childcare arrangements, and the necessity of secrecy from personal networks. It further entrenches a divide between those possessing the resources to seek care abroad and those who do not.^15^

Malta is a deeply patriarchal society, and the patriarchal structure is powerfully intertwined with the influence of the Catholic Church, which has historically worked to control women’s reproductive rights,^16^ culminating in a significant policy vacuum in sexual and reproductive health. For example, state-run family planning clinics, opened in 1982, were closed in the early 1990s due to pressure from the pro-life movement.^17^ The country’s first National Sexual Health Policy was only published in 2011 and was widely criticised by professionals as being outdated upon release and insufficient in its scope.^18^ For over a decade following its publication, there was only one under-resourced public sexual health clinic to serve the entire population,^18^ and a new national strategy was only launched in late 2024 after persistent pressure from activists.^19^

In the twenty-first century, the Church's influence over Maltese society began to decline substantially. The most significant political challenge to the Church's authority came in 2011, with the referendum on the introduction of divorce. Despite a vigorous “No” campaign led by the Church, the Maltese electorate voted in favour of legalisation, signalling a new era where religious moral authority no longer automatically translated into political reality.^12^ The outcome left the ruling Nationalist Party in disarray, appearing out of touch with the country's shifting moral compass. Conversely, the Labour Party, under a new younger leader, rebranded itself as a movement of progressives and moderates, championing LGBTIQ+ rights to appeal to younger voters.^20^ After making civil unions a key electoral pledge, the party secured a landslide victory in 2013, and in a remarkably short period, Malta transformed from a country with a poor record on LGBTIQ+ issues to being ranked first in Europe for its legal protections.^21^

Despite the broader trend towards social liberalisation, abortion remains a political taboo that both major parties are reluctant to address. When Malta joined the European Union, the government negotiated Protocol No. 7 of the Accession Treaty, which explicitly safeguards Malta's national abortion laws from EU influence.^22^ Following accession in 2004, the pro-life group Gift of Life unsuccessfully campaigned to entrench the abortion ban in the Constitution, an idea that continues to find support among some politicians.^10,21^ In the European Parliament, Maltese MEPs from both parties consistently oppose resolutions promoting abortion access.^21^

A historic challenge to this consensus came in 2021, when independent MP Marlene Farrugia, nearing the end of her term, tabled a private member’s bill to decriminalise abortion. The political establishment’s reaction was swift and unified. While the opposition Nationalist Party immediately declared it would vote against the bill,^23^ the governing Labour Party's response was more tactical. Prime Minister Robert Abela reiterated his party's anti-abortion stance but argued that a societal debate should precede any action by parliament.^24^ By refusing to debate it in parliament, the government effectively shelved the bill, reflecting a powerful cross-party agreement to avoid the issue. Even when the Prime Minister acknowledged in 2022 that his opinion had evolved, it was not seen as a commitment to legislative change.^15^

This history produced what Mark Harwood^21^ terms “Malta's Political Conundrum”: the paradox of a country celebrated as a leader in LGBTIQ +  rights, while enforcing one of the world's strictest abortion laws. Harwood argues that the state strategically embraced LGBTIQ+ rights to rebrand itself as modern, serving the Labour Party's political interests.^20^ However, this opening did not extend to abortion, which remained deeply controversial due to staunch public opposition.

The emergence of a vocal pro-choice movement in Malta is a recent phenomenon. A significant development was the successful campaign for emergency contraception in 2016. The campaign began with a grassroots movement on social media and culminated in a judicial protest filed on behalf of 102 women by the Women’s Rights Foundation (WRF), which successfully pressured the government to license emergency contraception for over-the-counter sale.^25^ This victory was a pivotal moment, paving the way for broader reproductive rights activism.^26^ In 2018, the WRF became the first local organisation to publicly adopt a pro-choice stance.^27^ The following year, the pro-choice coalition, Voice for Choice (VFC), was formed, and DFCM was set up.^28^ The formation of the VFC coalition marked a pivotal shift in activism, consolidating diverse organisations into a unified front to challenge the state’s absolute ban. Their activities ranged from political advocacy to public mobilisation, such as annual rallies. Beyond high-visibility protests, a robust infrastructure of local and international grassroots support has emerged to address the practical and emotional needs the state denies. These include the volunteer-operated FPAS in Malta, a helpline providing information on reproductive choices, the Abortion Support Network (ASN), which provides financial and logistical assistance for those forced to travel to access care, and the Abortion Doula Support Service (ADSS), which provides emotional and informational support to individuals navigating the process. Additionally, online platforms like Break the Taboo Malta (BTTM) offer anonymous spaces for sharing abortion experiences, effectively countering the state-enforced silence and the myth that Malta is an abortion-free territory.^28^

While the development of a pro-choice movement over the last decade has significantly increased public discussion surrounding abortion, abortion continues to be a subject of significant social stigma. At the same time, public opinion, while still predominantly anti-abortion, is showing signs of shifting. A 2022 survey found that while a majority oppose abortion, most (53.3%) do not agree with imprisoning women who have abortions.^29^ The lack of knowledge and the prevalence of misinformation remain significant barriers. Many health and social care professionals are themselves unclear about what information they are legally permitted to provide, operating in a climate of fear and wilful ignorance to protect themselves from potential repercussions.^28^ This systemic ignorance is perpetuated by a lack of official public health guidance and the absence of abortion-related training in medical and social care curricula.

Despite this situation, in recent years, the established method of travelling abroad for an abortion has been disrupted by a significant shift towards at-home, self-managed medical abortions using online telemedicine. A study of the online provider Women on Web (WoW) found that the number of abortion pill packages shipped to Malta increased more than sixfold between 2017 and 2021.^15^ This development fundamentally means Malta can no longer claim to be an abortion-free territory, as the procedure is now increasingly taking place within its borders, albeit outside the law.

## Methodology

This paper uses a qualitative case study design to analyse the events surrounding the Andrea Prudente case and the subsequent reform of Malta's abortion law. We chose this approach because it allowed us to investigate contemporary phenomena in their real-life context.^30^ We employed a prospective, real-time documentation strategy, recording and archiving media articles and institutional documents as they were published between June 2022 and June 2023. This included daily monitoring of all major national media portals, including independent and Church-affiliated outlets, as well as international coverage (e.g. *The Guardian, BBC, New York Times)*. Parliamentary proceedings were tracked via official transcripts and video recordings from the Parliament of Malta website.

Sources were selected for inclusion based on their direct thematic relevance to the case of Andrea Prudente, the parliamentary debate of Bill 28, or the specific social mobilisation efforts that arose in direct response to these events. We excluded general discussions of abortion in Malta that did not specifically reference these events, as well as social media content, unless it was a verified post from a key institutional actor. The final database of over 400 unique primary sources was built through chronological monitoring rather than retrospective keyword searches, ensuring no relevant items were omitted. All materials used are in the public domain; thus, no ethical approval or consent was required.

Our data collection was enhanced by our roles as active participant-observers. As lead activists and medical providers, we served as primary sources for local and international media, participating in debates, interviews, street protests, and parliamentary discussions, and authored position papers and opinion pieces that were published during this period. One of the authors (Professor Isabel Stabile) provided expert medical testimony during the Andrea Prudente proceedings before the Constitutional Court.

The analysis was conducted following a data analysis spiral through repeated rounds of organising and reading.^31^ This process utilised an abductive approach, allowing key themes to emerge inductively from the primary data while simultaneously being informed by the theoretical framework of reproductive governance. This approach enabled us to revisit the archive with a heightened degree of analytical distance three years after the initial events. Following Yin’s^30^ pattern-matching technique, we compared the empirical evidence against the predicted patterns inherent in our theoretical framework to identify recurring themes, summarised in Table 1, which organises the findings into four distinct chronological phases. While the analysis was informed by a total capture of over 400 unique primary sources, the news articles and citations in this article were selectively chosen as exemplars that most clearly illustrate new factual developments or diverse perspectives identified across the full dataset.

**Table 1.** Chronological–thematic framework (June 2022 – June 2023)

Phase & timeline	Analytical themes	Illustrative evidence
Phase 1: Medical Emergency (June-July 2022)	The chilling effect; biopolitics of the double effect; social mobilisation; transnational framing (Dobbs).	Doctors’ judicial protest; hospital “waiting” policy; medical evacuation to Spain; international media framing.
Phase 2: Legislative and Legal action (Sep – Dec 2022)	Strategic litigation; countermovement mobilisation; nationalist rhetoric; fetocentric biopolitics.	Filing of constitutional case; tabling of Bill 28; anti-choice mass protests; “Trojan horse” political discourse.
Phase 3: The court hearings (Jan – March 2023)	Contested scientific truths; institutional resistance; global vs local “regimes of truth”.	Courtroom as an arena for contesting state control; dispute over pPROM survival rates and “regimes of truth”; state advocate procedural hurdles.
Phase 4: Final Enactment (June 2023)	Political neutralisation; decoupling of law and access; hybrid discursive regimes.	Legislative dilution; state retrenchment; Act XXIII restricting access; three-doctor panel barrier.

As authors, we acknowledge our dual roles as researchers and key members within the Maltese pro-choice movement. Dr Andreana Dibben is a lead activist with WRF, while Dr Natalie Psaila Stabile and Professor Isabel Stabile are medical providers and co-founders of DFCM. While our roles as members of the Maltese pro-choice movement provided unique, real-time access to the sociopolitical and medical dynamics of the Prudente case, this insider status requires a reflexive acknowledgement of our positionality. We have sought to mitigate potential bias by grounding every claim in a verifiable audit trail of public documents and official records. We treated our own published articles, media appearances, and testimonies as primary data points, subjecting them to the same thematic scrutiny as the rest of the data.

A primary limitation of this study is its reliance on public records and digital archives, which may favour the perspectives of publicly active groups while underrepresenting the private experiences of residents who remain silent due to social stigma and fear. Furthermore, considering the decision to conclude the study period with the final enactment of Act XXII in June 2023, it is important to note that the constitutional case initiated by Andrea Prudente remains ongoing.* Consequently, this study cannot account for the final judicial resolution or its potential to further alter the reproductive landscape. Finally, while the two-year gap between the end period of data collection and analysis provided analytical distance, retrospective interpretation may emphasise certain patterns that were less apparent to us at the height of the crisis.

## Andrea Prudente case as a catalyst for mobilisation

The immediate aftermath of the publicity surrounding the Andrea Prudente case saw a surge of activism that directly challenged the state’s reproductive governance. A street protest was held in front of parliament,^32^ and activists galvanised their networks to bring international attention to the case. Within two days, 135 doctors filed a judicial protest arguing that the law compromised their ability to provide care.^33^ Andrea Prudente’s case revealed a critical and dangerous ambiguity in the application of the double effect principle,^†^ exemplifying how the abortion ban translated into clinical practice, creating a chilling effect where fear of legal repercussions overrides medical judgment and patient well-being. This situation is reminiscent of other contexts in which restrictive laws, even if not always enforced through prosecutions, create a climate of fear and obstruction that effectively denies care.^34,35^

Crucially, this mobilisation did not occur in a vacuum but was built upon the efforts of the nascent but organised pro-choice movement. This existing infrastructure of actors, operating in resistance to the official state position, was able to rapidly amplify Prudente’s story. Prudente was immediately connected with and guided by pro-choice doctors and lawyers. The couple's decision to seek support from DFCM for a second opinion and the subsequent recommendation for medical evacuation highlighted the tension between the state's monolithic control and alternative, rights-based approaches to care. This tension was further evidenced by the institutional enforcement of a dominant moral regime; the difficulties the couple faced in obtaining medical records and the reported hostility from staff illustrated this perfectly.^8^ The hospital's actions constituted an active exercise of power, prioritising a dominant moral regime over the immediate health and autonomy of the pregnant woman.

Adding to the global significance of her case, Andrea Prudente’s ordeal in Malta unfolded during the very same week that the United States Supreme Court overturned Roe v. Wade in its Dobbs v. Jackson Women’s Health Organisation decision.^36^ This major event in American jurisprudence sent shockwaves across the world, immediately intensifying the focus on countries with highly restrictive abortion laws. Suddenly, Malta was positioned as a stark example of the real-life consequences of absolute abortion bans at a moment when reproductive rights were being dramatically curtailed in the world’s most powerful democracy. International media attention was swift and intense, and reports in *The Guardian,* the *New York Times,* and the *BBC* framed Prudente’s situation as a clear violation of human rights.^37–39^ This global scrutiny was instrumental in transforming a domestic medical crisis into an international controversy, putting immense pressure on the Maltese government.

Another point to consider is how this mobilisation exposed the politics of silence that define Malta’s reproductive landscape. Significantly, it was a foreign individual with access to external support who dared to publicly challenge the state’s denial of care. Her situation stands in stark contrast to the experience of local Maltese women, who have historically remained silent.^40^ Within a restrictive governance framework, local women often lack the social and medical capital to speak up, fearing the pervasive stigma. This silence has allowed anti-choice actors to justify the absolute ban by claiming a lack of documented deaths,^41^ effectively ignoring the trauma of those who did not feel empowered to challenge the medical establishment.

## Constitutional court case and bill 28

In September 2022, Andrea Prudente, supported by lawyers from the WRF, initiated a constitutional court case against the Maltese government, specifically suing the Attorney General, the Health Minister, and the Parliamentary Secretary for Reforms and Equality. She argued that the state's blanket ban on abortion violated her fundamental human rights as protected by the Maltese Constitution and the European Convention on Human Rights, including the right to life, protection from inhuman and degrading treatment, respect for family life, and freedom from gender-based discrimination.^42^

This legal action, as well as the doctors’ judicial protest, represented a direct assault on the legislative controls that form the bedrock of Malta’s abortion ban, mirroring the tactics of strategic litigation seen in countries like Colombia, Brazil, and Argentina, where feminist movements have turned to the courts when legislative avenues are blocked.^5,6,43^ This strategy put pressure on Malta to align its governance with norms established by the European Court of Human Rights (ECtHR) and UN treaty bodies. The framing of women's rights as human rights is a key strategy for transnational advocacy networks, though its effectiveness can be limited when it clashes with deeply entrenched domestic norms and nationalist sentiments, as has been observed in post-Soviet contexts.^44^

In November 2022, the government tabled Bill 28, a draft amendment to permit abortion when a woman's life is at risk, or her health is in “grave jeopardy”.^45^ The government posited that Andrea Prudente’s case revealed a dangerous ambiguity in the application of the double effect principle. Despite her pregnancy being non-viable and posing a high risk of life-threatening sepsis, doctors in Malta felt unable to intervene, highlighting that in practice, medical professionals may interpret the law as preventing them from acting until the threat to the woman's life is imminent, rather than intervening to prevent a potential and likely danger like sepsis. Consequently, the government argued that the theoretical application of the principle of double effect can conflict with the practical realities of medical emergencies, leaving patients in perilous situations and doctors in a legal grey area.^46^^,^^47^

The initial proposal of Bill 28, to allow abortion when a woman's health was at grave risk, was a clear concession to the pressure from pro-choice advocates and the international community. The government was likely attempting to pre-empt an unfavourable ruling from the Constitutional Court or eventually the ECtHR, thereby managing its position within a broader European rights framework. This mirrored the situation in Ireland, where the government had faced an adverse ruling in *A, B, and C v. Ireland* for failing to provide a clear and accessible procedure for lawful abortions.^48^ The initial bill was met with cautious optimism by pro-choice activists. While falling short of decriminalisation, it signalled a potential shift in the state's governance, acknowledging for the first time that the protection of a pregnant person's health could, in principle, override the absolute protection of a fetus.

In parliamentary speeches, Prime Minister Robert Abela and Health Minister Chris Fearne consistently presented the amendment as a clarification of existing medical practice, not as an introduction of abortion.^49,50^ They emphasised that the bill was about saving women's lives and protecting doctors from prosecution, deliberately avoiding the language of “rights”, “choice”, or “autonomy”. This reflected a political calculation that a debate framed around health and safety was more winnable than one framed around feminist principles or a rights-based discourse.

## Countermovement and suppression

The government's proposal was immediately met with fierce and highly organised opposition, demonstrating the power of other actors within the reproductive governance framework. A well-organised countermovement, led by conservative civil society groups and heavily supported by the Catholic Church, mobilised to reassert its moral injunctions. This coalition leveraged its significant institutional power and cultural influence to frame any liberalisation of abortion law as a threat to Maltese identity and morality, a tactic common in deeply Catholic nations like Poland and pre-repeal Ireland.^51^ The strategies employed were classic mechanisms of reproductive governance, a familiar pattern that the same actors had activated to oppose previous progressive developments, including the morning-after pill, assisted reproductive technologies, and equality legislation. The response to Bill 28, however, was significantly more intense. Mass protests were organised, echoing the pro-life campaigns in Ireland that relied on a strong emotional connection to a morally superior national identity^13,^^52^^,^^53^ Eighty academics, including legal and medical experts, published a position paper, tabled in parliament by Opposition MPs, opposing the amendment since the term “health” could allow a woman with serious mental health conditions to access abortion.^54^ The President of Malta at the time, Dr George Vella, a medical doctor, known for his anti-abortion views, indicated he was prepared to resign rather than sign the abortion law amendment and broke with tradition by speaking about the amendment during the Republic Day speech.^55^^,^^56^ The Catholic Bishops of Malta sent letters to all MPs urging them to oppose the “threat to health” clause.^57^

The Life Network Foundation, an organisation that forms part of a wider transnational anti-gender movement that strategically targets sexual and reproductive health and rights to advance a conservative agenda,^58,59^ led a coalition of 45 mostly church-affiliated organisations in calling for the bill to be restricted to cases of threat to life only. Former President Marie-Louise Coleiro Preca publicly endorsed this position.^60^ Catholic schools also sent letters to all parents urging them to join the mass protests.^61^ The intensity of the response was itself significant, suggesting that abortion held more weight within Maltese reproductive governance than previous reforms, with Malta considered a last frontier in Europe. In response, MEP Cyrus Engerer gathered signatures from 108 MEPs and 55 parliamentarians from 15 other national parliaments in support of the original bill, congratulating Malta for taking a step to protect women's healthcare.^62^ Over 150 academics signed a counter-position paper supporting the original bill, which was tabled in Parliament by a government MP.^63^

The countermovement disseminated a narrative that aimed to discredit pro-choice activists, accusing them of a hidden agenda and misrepresenting the proposed amendment as a gateway to abortion-on-demand. Former Nationalist MP Jason Azzopardi posted on Facebook, claiming without evidence that Ms Prudente's case was a “conspiracy” to introduce abortion to Malta. He alleged that she was brought to Malta to create a controversy over the termination of a pregnancy due to a pretended danger. Blogger and history professor Simon Mercieca published a similar story. In December 2022, Ms Prudente filed libel suits against both men.^64^ The Opposition Leader, in a widely criticised speech in parliament, articulated the core of the opposition's argument: that the proposed amendment was “abortion through the window”, an attack on Maltese values that would lead to the same path as other countries. He argued that the term “health” was dangerously ambiguous and would be exploited to justify abortion on demand, framing the debate as a defence of life. During his speech, he appeared to mock Prudente, dismissively referring to her throughout as “that American woman”, using a play on her name to suggest that the doctors who treated her had been more “*prudenti”* (prudent) than she was, questioning her motives for coming to Malta, while arguing that her life had never truly been at risk.^65^ Other Opposition MPs went further, invoking the nation's soul, civilisational decline, and Malta's moral identity, framing the vote as a historic crossroads between a culture of life and a culture of death.^66^^,^^67^ Prime Minister Abela responded by labelling Grech’s speech as “shocking” and “misogynistic”, attempting to position his government as the defender of women against a backwards-looking opposition.^68^ However, the substance of the debate remained firmly within the parameters set by the conservative countermovement. The discussion was dominated by arguments about the “sanctity of life from conception” and the potential for the “health” clause to be abused. The rights and autonomy of the pregnant person were almost entirely absent from the mainstream political discourse, a phenomenon described as the fetocentric framing of abortion law reform in Ireland.^69^ A few pro-choice legislators argued from a rights-based framework, emphasising bodily autonomy and framing abortion as essential health care.^70–72^ They invoked the proportionality principle, arguing that a total ban on abortion in cases like Prudente's is disproportionate because it completely sacrifices the woman's fundamental rights to life, health, and dignity for the sake of protecting a non-viable fetus.^5^ However, these arguments were pitted against powerful narratives centred on fetal rights and religious morality.

The politicisation of the Prudente case was a clear manifestation of reproductive governance in the public sphere. The claims of a “conspiracy” and the mockery of Andrea Prudente were strategic political manoeuvres designed to enforce a specific moral order. By questioning Ms Prudente's motives and framing her case as a threat to the nation, they employed nationalist and moralising rhetoric to delegitimise her and the broader call for reform.^51^ Prime Minister Abela's condemnation of these remarks as misogynistic indicates the presence of competing political logics, yet the overall climate remained one where a woman's medical crisis was subordinated to a national political and moral drama. As Morgan^73^ describes, this local opposition can be seen as part of a global anti-abortion movement that strategically invokes national sovereignty and tradition to resist international human rights norms. The rhetoric used in Malta was strikingly similar to that seen in other contexts, such as Macedonia, where nationalist governments have instrumentalised reproductive rights as part of a larger project of re-traditionalisation and ethnic nation-building.^74^

## Debate over medical evidence and professional conduct

The court hearings, which began on 6 January 2023, brought the details of Andrea Prudente’s medical care into public view. On one side, Dr Muscat Baron and Dr Alberto Vella, representing Mater Dei Hospital, testified that Ms Prudente was “never in danger of dying” and claimed there was a possibility the fetus could survive, citing a study allegedly showing a 79.2% survival rate for pPROM before 20 weeks and a concurrent case of pPROM at 13 weeks where the pregnancy continued for three months and resulted in a live birth. They stated that the hospital’s policy is to wait until the mother's life is endangered and attributed Andrea Prudente’s desire for discharge to her wish to “terminate rather than wait”.^75^

The human cost of this model was exposed through the testimony of Jay Weeldryer. Recounting his partner's subsequent diagnosis of severe post-traumatic stress disorder (PTSD), he described an atmosphere of uncertainty among the staff as to “what the hell to do with us”, alongside their battle to obtain medical records.^76^ A powerful counter-narrative was also introduced through the testimony of Professor Isabel Stabile (one of the authors) of DFCM. Arguing the pregnancy was “doomed to fail”, she reviewed the medical records to highlight signs of placental bleeding, severe lack of amniotic fluid and a slow fetal heart rate. Citing international guidelines and a supporting letter from the International Federation of Gynaecology and Obstetrics (FIGO), she asserted the risk of sepsis and haemorrhage to the woman far outweighed the negligible chance of survival for the fetus.^77,78^

The conflicting testimonies sparked a public debate over medical evidence. DFCM issued a statement refuting the 79% survival rate claim made in court by Muscat Baron, clarifying that the Israeli study he referenced – Herzlich et al.,^79^ – included only 24 cases of pPROM between 17 and 23 weeks, and that none of the fetuses in that study where rupture occurred before 19 weeks survived to discharge. As Ms Prudente's rupture occurred at 15 weeks, the study did not apply to her clinical case and, in fact, argued against the possibility of survival.^80^ The Israeli study was contrasted with a 2016 Irish study from a similarly restrictive climate,^81^ which showed an infant survival rate of less than 5% for mid-trimester pPROM.^82^ The case hearings also led to accusations of professional misconduct, with Doctors for Life accusing Prof Stabile of placing “undue pressure” on the medical team. DFCM vehemently denied this, condemning the accusation as an “attack on patient autonomy and their right to seek a second opinion”.^83^

Andrea Prudente's constitutional court case effectively transformed the courtroom into an arena for contesting Malta's reproductive governance, framing the abortion ban as a violation of fundamental human rights that challenges the nature of women's citizenship.^84^ The hospital’s testimony represented the state's official position, reflecting a form of governance where intervention is permitted only at the threshold of death, a stance that, drawing on Morgan,^73^ aligns with an anti-globalist approach that resists international human rights norms on reproductive rights. In contrast, Professor Stabile's testimony sought to shift the “regime of truth”^8^ from a local, legal framework to a global, medico-scientific one. The public dispute over medical evidence was a struggle to define scientific and moral justifications, demonstrating how “facts” are mobilised to legitimise pre-existing positions in abortion politics.^85^ Furthermore, the accusation of “undue pressure” can be seen as an attempt by defenders of the status quo to police professional boundaries. The debate highlighted the fundamental clash between a paternalistic model of state governance and a rights-based model centred on patient autonomy.

The legal battle also encountered significant procedural hurdles that further tested the plaintiff. Citing the need to protect Ms Prudente’s well-being, considering her trauma, her lawyer successfully requested that the case continue behind closed doors. However, further barriers arose from the state's side. In a move that delayed the proceedings, the State Advocate filed a last-minute objection to Andrea Prudente testifying via video conference from the United States, an issue which had to be resolved by the court.^86^

## Final legislation and its impact

A significantly altered and “watered-down” version of Bill 28 was published in June 2023. It was clear that the state had capitulated to the immense pressure from the conservative bloc, including medical unions and the threat by the President of Malta, which could have sparked a constitutional crisis.^87^ Pro-choice activists attended the committee hearing in a final bid to convince legislators of the potential perilous consequences of the watered-down bill, but their contributions were largely ignored.^88^

The final legislation, Act No. XXII of 2023, passed in June 2023, permits a termination of pregnancy only when the person's life is at “immediate risk”. In cases of a medical complication that places the person in “grave jeopardy which may lead to death”, a termination is only allowed following the unanimous approval of a panel of three registered specialists (two obstetricians/gynaecologists and a specialist in the relevant complication), adding a significant bureaucratic barrier.^89^ The new law was met with dismay by pro-choice advocates.^90^ Medical specialists noted that the amendment was so restrictive that it would not have helped Andrea Prudente and might even create further delays.^91^ Ms Prudente herself described the law as “empty and heartless”, stating it would not have saved her from her ordeal.^92^ Conversely, the Catholic Church and anti-choice groups welcomed the amendment, thanking the government for listening to their concerns.^90^

This outcome is a powerful example of how reproductive governance can function to absorb and neutralise challenges. The state enacted a reform, but one so restrictive that it creates new legal and procedural barriers. The resulting legislation in Malta reveals both striking similarities to and significant differences from its international counterparts. Much like the early, failed Irish model of the Protection of Life During Pregnancy Act 2013, the Maltese reform prioritises the protection of doctors from prosecution over the timely care of patients by mandating unworkable multi-doctor panels.^7^ However, while the Irish juncture eventually led to the 2018 repeal of the Eighth Amendment and the return of reproductive agency to women,^69^ Malta’s outcome remains a form of “conditional legalisation”.^93^ On the surface, this change appears to be a reform, but in practice it creates painstaking bureaucratic procedures. It is a situation that creates a chilling effect on providers, as Barıs¸^34^ describes in the context of Turkey, and ensures that meaningful access remains elusive.

This outcome stands in sharp contrast to the Colombian model, where the Court’s Decision C-355/2006 incorporated international human rights law to declare absolute bans unconstitutional, thereby dignifying women by exhibiting a deep understanding of their lived medical experiences.^5^ Furthermore, unlike Argentina’s 2020 legalisation, which was the product of decades of social decriminalisation and the framing of abortion as a matter of democratic citizenship, the Maltese state “neutralised” the critical juncture by absorbing the challenge into a restrictive law that largely preserves the status quo.^6^

## Conclusion

The Andrea Prudente case was a powerful catalyst that forced a crack in Malta's absolute abortion ban. It triggered significant social mobilisation that successfully compelled the state to act. However, the complex power struggle that ensued between competing actors revealed that the dominant mode of reproductive governance in Malta remains one of legal prohibition and moral condemnation, creating a stark conflict between state power and an individual’s right to health and citizenship.^84^

The state, caught between a pro-choice movement pushing for change and a powerful conservative establishment determined to prevent it, passed a law that appeared responsive on the surface, while fundamentally preserving the existing restrictive regime. The final law is clear evidence of the enduring power of religious and moral injunctions within Malta's reproductive governance. It demonstrates how legal reform can be decoupled from the lived realities of those seeking care, creating what has been described as a “hybrid discursive regime” in which a nominal legal right exists alongside immense practical barriers.^94^ The Maltese outcome reflects a victory for the nationalist, pro-sovereignty approach over the universalist human rights strategy that has led to more substantial reforms in countries such as Argentina and Colombia.^6,73^ The framing of abortion as a threat to the nation's character echoes the Irish experience and demonstrates the deep-seated cultural attachments that can thwart rights-based reform.^13^

While the legislative outcome was a deep disappointment for advocates of reproductive justice, the Prudente case has irrevocably fractured the previous politico-legal consensus. It demonstrated that the governance framework, though deeply entrenched, can nonetheless be challenged. While legal reform has been limited, it set a significant precedent for future challenges and strengthened the pro-choice movement’s pursuit of abortion decriminalisation and comprehensive reproductive health care for all.

### Postscript

Significant developments following our study period have brought the risks of self-managed abortion into public discourse. In 2025, a woman who sought hospital care for complications following a self-managed abortion was reported to the police by emergency healthcare providers, subsequently receiving a suspended prison sentence.^95^ In March 2026, another woman received a suspended sentence in a similar case, again following a report made by doctors at Mater Dei Hospital.^96^ These cases confirm that the medical environment remains a site of criminal risk. The Malta Medical Council (MMC) responded in April 2026, issuing a memo clarifying that doctors are not obliged to report women presenting with abortion complications to the police.^97^ In January 2026, the Prime Minister coupled his assurance that he would “not let a woman go to prison” for seeking an abortion with the definitive caveat that the Labour Party’s next electoral manifesto would not include any mention of legal reform, effectively bypassing an electoral mandate for decriminalisation.^98^ Following the March 2026 sentencing, the Prime Minister again signalled a willingness to seek Cabinet review of the suspended sentence.^99^ Meanwhile, Labour won the general election in May 2026, where the issue of abortion was conspicuously absent from public discourse. These developments validate our conclusion that the state’s primary strategy remains one of neutralisation. It offers a language of empathy for the individual while preserving the legal regime that enables the reporting and prosecution of women. Notably, the MMC’s memo extends this neutralisation into the clinical sphere, effectively relieving pressure on medical providers without any legislative reform. This suggests that the hybrid discursive regime identified in 2023 continues to characterise the Maltese state, where leadership acknowledges the injustice of the law in rhetoric but is unwilling to act because the political risk is considered too high.

### Addendum

Since the acceptance of this paper, the Civil Court of Malta (First Hall, Constitutional Jurisdiction)^100^ delivered its judgment in *Andrea Prudente v. the State of Malta* on 1 July 2026, ruling against the applicant and holding that her rights were not violated when doctors refused to terminate her pregnancy. The judgment was criticised by Voice for Choice both for its outcome and its framing. The judge decreed that Prudente had been “used by so-called pro-choice people” to advance their agenda without regard for her emotional state. In a press release, Voice for Choice condemned this statement as an insult to Prudente's autonomy and noted that the judgment disregarded submissions from the International Federation of Gynaecology and Obstetrics regarding the elevated risk of fatal infection in such cases.^101^ The European Centre of the International Council of Women announced its intention to seek an examination of the judgment's compatibility with EU treaty obligations and the Charter of Fundamental Rights.^102^ The outcome of the constitutional case confirms and deepens the central argument of this paper.

## Supplementary Material

List of Documents used for the analysis_Updated_18062026_.docx

ZRHM-2025-0250_Appendix.Full list of sources_.xlsx

## Footnotes

* Since acceptance of this paper, judgment was delivered in this case on 1 July 2026. The Court ruled against the applicant. The judgment is addressed in the Addendum.

† The doctrine of double effect allows for medical interventions necessary to save a woman's life, even if they result in the unintended death of the fetus. The key distinction is the absence of criminal intent (*mens rea*) to procure a miscarriage; the primary goal is to treat the pregnant person.^5^

## Provenance statement

This article was not commissioned and went through external peer review.
