# Supplementary material for: Andrea Prudente case: a case study on social mobilisation, reproductive governance, and the politics of abortion reform in Malta
Source: Sex Reprod Health Matters. 2026 Jul 29;33(1):2697378. doi: 10.1080/26410397.2026.2697378 (PMC13573501; doi:10.1080/26410397.2026.2697378)
Supplement: Supplementary file 1 [file ZRHM_A_2697378_SM6207.docx]

**List of material used for the analysis**

Abela, R. (2022, November 29). [Speech on Bill No. 28]. In *House of Representatives: Plenary Session 062.* Parliament of Malta. <https://parlament.mt/en/14th-leg/plenary-session/ps-062-29112022-0400-pm/>

Agius, E., Aquilina, K. & Attard, J. (2022, November). *An act to further amend the Criminal Code, Cap. 9. Bill 28: A position paper. https://knisja.mt/wp-content/uploads/2022/11/POSITION-PAPER-AN-ACT-to-further-amend-the-Criminal-Code-Cap.-9.pdf?_ga=2.190738284.630688080.1757321691-831595505.1757321690*

Agius, M. (2022). Andrea Prudente: constitutional case filed over abortion law. *Malta Today.* <https://www.maltatoday.com.mt/news/court_and_police/118872/andrea_prudente_constitutional_case_filed_over_malta_abortion_law>

Azzopardi, C. (2022, November 30). [Speech on Bill No. 28]. In House of Representatives: Plenary Session 064. Parliament of Malta. https://parlament.mt/en/14th-leg/plenary-session/ps-064-30112022-0400-pm/

Azzopardi, K. (2023, 26 June). Uncomfortable government MPs face ‘betrayed’ pro-choice activists in parliamentary committee. *Malta Today.* <https://www.maltatoday.com.mt/news/national/123660/uncomfortable_government_mps_face_betrayed_prochoice_activists_in_parliamentary_committee_>

Balzan, J. (2023, January 10). Doctors for Life urge MPs to ‘protect the unborn child against intentional and direct harm’. *Newsbook.* <https://newsbook.com.mt/en/doctors-for-life-urge-mps-to-protect-the-unborn-child-against-intentional-and-direct-harm/>

Bill No. 28 (2022). *A Bill to amend the Criminal Code, Cap. 9*, Bill No. 28, Parliament of Malta, 14th Legislature. (2022). <https://parlament.mt/media/119611/bill-28-criminal-code.pdf>

Borg, N. (2023, June 25). New abortion bill would make no difference in a case similar to Andrea Prudente. *Times of Malta.* https://timesofmalta.com/article/new-abortion-bill-make-no-difference-prudente-case.1039938

Brincat, E. (2023a, February 5). Andrea Prudente felt trapped in Malta despite being told her ‘baby was lost’. *Times of Malta.* https://timesofmalta.com/article/andrea-prudente-felt-trapped-malta-despite-told-baby-lost.1011666

Brincat, E. (2023b, March 8). Medical professor says Prudente’s pregnancy ‘doomed to fail.’ *Times of Malta.* <https://timesofmalta.com/article/medical-professor-says-prudentes-pregnancy-doomed-fail.1018131>

Brincat, E. (2023c, March 8) State advocate objects to Andrea Prudente testifying by video conference. *Times of Malta.* https://timesofmalta.com/article/state-advocate-objects-andrea-prudente-testifying-video-conference.1018107

Brincat, E. (2023d, January 6). There was a ‘possibility’ Prudente baby could live, doctor tells court. *Times of Malta.* https://timesofmalta.com/article/possibility-prudente-baby-live-doctor-tells-court.1005727

Buttigieg, R. (2022, November 28). [Speech on Bill No. 28]. In *House of Representatives: Plenary Session 061.* Parliament of Malta. <https://parlament.mt/en/14th-leg/plenary-session/ps-061-28112022-0400-pm/>

Calleja, C. (2022a, June 22). ‘Don’t risk her life’, appeals partner in plea for abortion after miscarriage. *Times of Malta.* [*https://timesofmalta.com/article/came-babymoon-abortion-couple-say.963500*](https://timesofmalta.com/article/came-babymoon-abortion-couple-say.963500)*.*

Calleja, C. (2022b, July 5). ‘Our priority is: mother first’: the medical procedure amid Malta’s abortion ban. *Times of Malta.* https://timesofmalta.com/article/explained-medical-procedure-amid-malta-s-abortion-ban.965656

Calleja, C. (2022c, June 24). ‘Terrifying ordeal is over’: woman denied abortion in Malta treated in Spain. *Times of Malta.* [*https://timesofmalta.com/article/a-terrifying-ordeal-over-woman-denied-abortion-malta-treated-spain.964028*](https://timesofmalta.com/article/a-terrifying-ordeal-over-woman-denied-abortion-malta-treated-spain.964028)*.*

Calleja, C. (2022c, December 1). US couple file libel suits over claims they conspired to introduce abortion. *Times of Malta.* <https://timesofmalta.com/article/us-couple-file-libel-suits-claims-conspired-introduce-abortion.998695>

Carabott, S. (2022, June 27). 135 doctors sign juidicial protest asking for review of Malta’s abortion ban. *Times of Malta.* <https://timesofmalta.com/article/135-doctors-sign-judicial-protest-asking-review-maltas-abortion-ban.964433>

Conry, J. (2023, February 1). *Scientific opinion of FIGO in the Constitutional Court case of Andrea Prudente against the Maltese state.* [*https://www.doctorsforchoice.mt/post/scientific-opinion-of-figo-in-the-constitutional-court-case-of-andrea-prudente-against-the-maltese-s*](https://www.doctorsforchoice.mt/post/scientific-opinion-of-figo-in-the-constitutional-court-case-of-andrea-prudente-against-the-maltese-s)

*Criminal Code (Amendment) Act, 2023*, Act No. XXII of 2023 of the Laws of Malta. <https://legislation.mt/eli/act/2023/22/eng>

Cutajar, R. (2022, November 29). [Speech on Bill No. 28]. In *House of Representatives: Plenary Session 062.* Parliament of Malta. https://parlament.mt/en/14th-leg/plenary-session/ps-062-29112022-0400-pm/

De Battista, R. (2022, November 28). [Speech on Bill No. 28]. In *House of Representatives: Plenary Session 061.* Parliament of Malta. <https://parlament.mt/en/14th-leg/plenary-session/ps-061-28112022-0400-pm/>

Diacono, T. (2022a, 23 June). A Maltese Woman Was Just Forced To Carry Unviable Pregnancy For Six Weeks, Gynaecologist Warns. *Lovin Malta.* <https://lovinmalta.com/news/a-maltese-woman-was-just-forced-to-carry-unviable-pregnancy-for-six-weeks-gynaecologist-warns/>.

Diacono, T. (2022b, 1 December). Maltese church school urges students and parents to sign anti-abortion bill petition and attend protest. *Lovin Malta.* <https://lovinmalta.com/news/maltese-church-school-urges-students-and-parents-to-sign-anti-abortion-bill-petition-and-attend-protest/>

Dibben, A. (2023, January 27). Prudente and critics: a reply – Andrea Dibben. *Times of Malta.* <https://timesofmalta.com/article/prudente-critics-reply-andrea-dibben.1009452>

Dibben, A. Et (2022, December). *Statement by other academics in support of Bill 28.* https://cdn-others.timesofmalta.com/b1b2d95ec2888bc0421f8d61e98c3e27069e616b.pdf

Doctors for Choice Malta. (2023, January 12). *Study used to claim Andrea Prudente’s fetus had a chance actually showed the exact opposite.* <https://www.doctorsforchoice.mt/post/study-used-to-claim-andrea-s-fetus-had-a-chance-actually-showed-the-exact-opposite?fbclid=IwAR09ZTI4EGJhlq3Z9nfO26kPFnktqiT36T2dJKGXt9D92wAv9M-1mbCQ4cY>

Fearne, C. (2022, November 28). [Speech on Bill No. 28]. In *House of Representatives: Plenary Session 061.* Parliament of Malta. <https://parlament.mt/en/14th-leg/plenary-session/ps-061-28112022-0400-pm/>

Gravino, G., & Stabile, I. (2023). Maternal mortality: Nothing but the truth. *MaltaToday.* <https://www.maltatoday.com.mt/comment/opinions/120364/maternal_mortality_nothing_but_the_truth_gilbert_gravino_isabel_stabile>

Grech, B. (2022, November 30). [Speech on Bill No. 28]. In *House of Representatives: Plenary Session 064.* Parliament of Malta. <https://parlament.mt/en/14th-leg/plenary-session/ps-064-30112022-0400-pm/>

Fenech Adami, B. (2022, November 30). [Speech on Bill No. 28]. In House of Representatives: Plenary Session 064. Parliament of Malta. https://parlament.mt/en/14th-leg/plenary-session/ps-064-30112022-0400-pm/

Magri, G. (2022a, June 22). Activists gather to call for abortion law change. *Times of Malta.* [*https://timesofmalta.com/article/activists-call-decriminalisation-abortion-mother-life.963602*](https://timesofmalta.com/article/activists-call-decriminalisation-abortion-mother-life.963602)

Magri, G. (2022b, December 13). ‘Bullies dancing on my daughter’s grave’: Prudente’s partner reacts to claims. *Times of Malta.* https://timesofmalta.com/article/bullies-dancing-grave-daughter-prudentes-partner-reacts-claims.1001329

Magri, G. (2022c, December 7). ‘Candles of life’ vigil held outside parliament in another anti-abortion protest. *Times of Malta.* https://timesofmalta.com/article/candles-life-vigil-parliament-anti-abortion-protest.1000141

Magri, G. (2022d, December 4). Thousands gather in Valletta to protest abortion law reform. *Times of Malta. https://timesofmalta.com/article/thousands-demand-stop-abortion-amendments-valletta-rally.999424*

Magri, G. (2023, June 23). Pro-life relief, pro-choice dismay: reactions to changed abortion bill. *Times of Malta.* [*https://timesofmalta.com/article/prolife-relief-prochoice-dismay-reactions-changed-abortion-bill.1039527*](https://timesofmalta.com/article/prolife-relief-prochoice-dismay-reactions-changed-abortion-bill.1039527)

Martin (2022, November 16). Abortion to be allowed when woman’s health is at serious risk. *Times of Malta.* https://timesofmalta.com/article/abortion-allowed-mothers-health-serious-risk.995190

Meilak, N. (2021, May 14). Labour Party takes no stand on decriminalisation of abortion. *Malta Today.* <https://www.maltatoday.com.mt/news/national/109621/labour_party_takes_no_stand_on_decriminalisation_of_abortion>

Meilak, N. (2022). PM hits out at Bernard Grech over ‘misogynistic’ speech in Parliament. *Malta Today.* <https://www.maltatoday.com.mt/news/national/120123/watch_pm_hits_out_at_bernard_grech_over_misogynistic_speech_in_parliament>

Sansone, K. (2022b, November 29). Over 100 MEPs support Maltese government abortion amendments. *Malta Today.* https://www.maltatoday.com.mt/news/national/120085/more_than_100_meps_support_government_abortion_amendments

Sansone, K. (2023, June 25). George Vella’s no to abortion Bill forced government’s hand. *Malta Today.* https://www.maltatoday.com.mt/news/national/123618/george_vellas_no_to_abortion_bill_forced_governments_hand

Times of Malta. (2021). PN can never back the decriminalisation of abortion – Bernard Grech. *Times of Malta.* <https://timesofmalta.com/article/pn-can-never-back-the-decriminalisation-of-abortion-bernard-grech.871411>

Times of Malta (2022a, November 21). Bill allowing abortion if woman’s health in ‘grave jeopardy’ is presented. *Times of Malta. https://timesofmalta.com/article/amendments-abortion-laws-tabled-parliament.996234*

Times of Malta. (2022b, December 2). Bishops on abortion: ‘It’s science, life starts at conception’. *Times of Malta.* https://timesofmalta.com/article/bishops-abortion-its-science-life-starts-conception.998998

Times of Malta (2022c, November 29). Former president Coleiro Preca joins calls to redraft abortion law changes. *Times of Malta.* <https://timesofmalta.com/article/former-president-coleiro-preca-calls-redraft-abortion-law-changes.998223>

Times of Malta (2022d, December 5). Over 100 academics support proposed changes to Malta’s abortion laws. *Times of Malta.* https://timesofmalta.com/article/100-academics-support-proposed-changes-maltas-abortion-laws.999646

Times of Malta. (2022e, November 22). Pregnancy could be terminated for mental health reasons under new law – experts. *Times of Malta.* <https://timesofmalta.com/article/pregnancy-terminated-mental-health-reasons-new-law-experts.996483>

Times of Malta. (2023, June 29). Couple whose pregnancy sparked abortion reform slam ‘empty, heartless’ law. *Times of Malta.* https://timesofmalta.com/article/couple-pregnancy-sparked-abortion-reform-slam-empty-heartless-law.1040688

Zammit, M.L. (2022a, November 6). Law to protect doctors, women who terminate pregnancy to safeguard health – PM. *Times of Malta.* <https://timesofmalta.com/article/doctors-women-protected-terminating-pregancy-safeguard-health-pm.992720>

Zammit, M.L. (2022b, December 13). President breaks tradition to raise abortion amendment in Republic Day speech. *Times of Malta. https://timesofmalta.com/article/president-breaks-tradition-raise-abortion.1001296*

Zammit M. L. (2022c, November 30). President ‘prepared to resign’ over abortion law amendment. *Times of Malta.* <https://timesofmalta.com/article/president-prepared-resign-abortion-law-amendment.998406>
